# ‘TLDetect’: AI-Based Application for Detection and Correction of Anomalous TLD Glow Curves

**DOI:** 10.3390/s24216904

**Published:** 2024-10-28

**Authors:** Gal Amit, Roy Vagerman, Oran Revayev

**Affiliations:** 1 Dosimetry Section, Soreq Nuclear Research Center, Yavne 8180000, Israel; 2 Systems Development Division, Soreq Nuclear Research Center, Yavne 8180000, Israel

**Keywords:** ionizing radiation dosimetry, thermoluminescence dosimetry, glow curve analysis, anomaly detection, dose assessment, machine learning

## Abstract

This research reviews a novel artificial intelligence (AI)-based application called TLDetect, which filters and classifies anomalous glow curves (GCs) of thermoluminescent dosimeters (TLDs). Until recently, GC review and correction in the lab were performed using an old in-house software, which uses the Microsoft Access database and allows the laboratory technician to manually review and correct almost all GCs without any filtering. The newly developed application TLDetect uses a modern SQL database and filters out only the necessary GCs for technician review. TLDetect first uses an artificial neural network (ANN) model to filter out all regular GCs. Afterwards, it automatically classifies the rest of the GCs into five different anomaly classes. These five classes are defined by the typical patterns of GCs, i.e., high noise at either low or high temperature channels, untypical GC width (either wide or narrow), shifted GCs whether to the low or to the high temperatures, spikes, and a last class that contains all other unclassified anomalies. By this automatic filtering and classification, the algorithm substantially reduces the amount of the technician’s time spent reviewing the GCs and makes the external dosimetry laboratory dose assessment process more repeatable, more accurate, and faster. Moreover, a database of the class anomalies distribution over time of GCs is saved along with all their relevant statistics, which can later assist with preliminary diagnosis of TLD reader hardware issues.

## 1. Introduction

**Figure 1:**
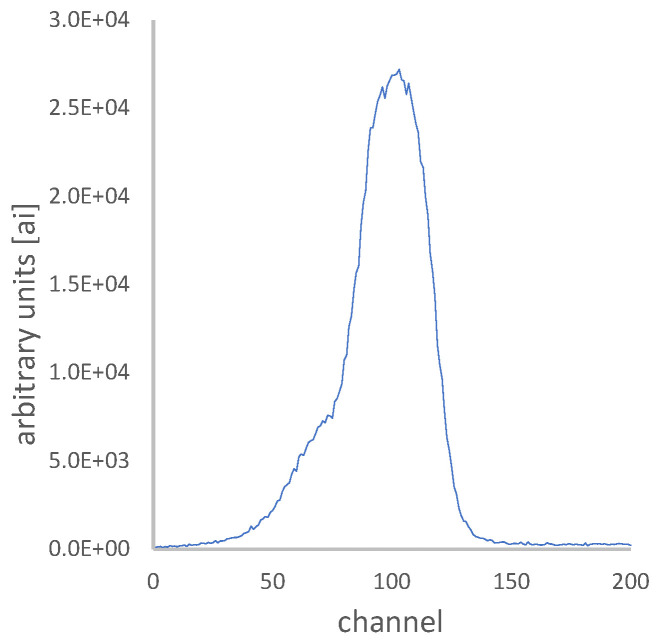
An example of a TLD glow curve, which is the measured output current of the thermoluminescence as a function of the time heating rate. The integral of this glow curve is proportional to the ionizing radiation that was absorbed in the TLD while it was worn by the radiation worker.

Radiation workers worldwide are being monitored for occupational ionizing radiation exposure by national dosimetry services. The ionizing radiation exposure routine monitoring cannot be underestimated, since the safety of workers is at stake. For this reason, all over the world, across medical, industrial, and nuclear sectors, all workplaces try to maintain high quality and precision exposure measurements of eye lens and whole-body doses [1,2,3,4,5,6,7,8,9,10,11,12,13]. Thermoluminescence dosimetry is a widespread technology for performing personal occupational dosimetry, which measures X-ray, gamma, beta, and thermal neutron radiation. The dose is estimated using a reader that heats the thermoluminescent dosimeter (TLD) element and reads the current initiated by the emitted photons. This current is proportional to the radiation exposure. The measured output current of the thermoluminescence (TL) as a function of the time heating rate is called a glow curve (GC), and its integral is proportional to the radiation absorbed by the dosimeter. Figure 1 shows a representative example of a GC.

The research was conducted using a Thermo Harshaw 8800 reader (Thermo Fisher Scientific Inc., Waltham, MA, USA) and TLD cards of type LiF:Mg, Ti. The reader uses the default time–temperature profile (TTP) of 25 °C per second in order to heat the TLD elements. Cards of type LiF:Mg, Ti possess a complex GC containing at least ten different glow peaks (GPs) ranging from room temperature to 400 °C. The main GP, referred to as peak number five, used for dosimetry in this card type at this TTP appears at about 205 °C.

The accurate estimation of the absorbed ionizing dose in radiation workers is of great importance since it is purely a matter of radiation safety for workers. For this reason, the GC examination and correction should be done by external dosimetry laboratories (EDLs) with great precision, accuracy, and repeatability. For this reason a lot of efforts are invested on the analysis of GCs quality prior to the dose estimation in a method that is as automated as possible [14].

Over the past two decades, numerous computerized glow curve analysis (CGCA) techniques have been developed and studied [15,16,17,18], as well as deconvolution methods to reveal various TLD glow peaks [19,20]. Some have been employed to determine parameters of kinetic models [21], while others have focused on improving the accuracy of instrumental background assessment for low-dose assessments or implementing internal quality criteria to detect anomalies [22,23]. Additional applications of CGCA have aimed at identifying specific anomalies within glow curves [24].

One of the earliest applications of machine learning (ML) for TL dosimetry [25] used an artificial neural network (ANN) to estimate doses in LiF:Mg:Ti-based four-element dosimeters. More recently, researchers have demonstrated the applicability of ML algorithms in TL dating [26], classification of thermoluminescence features of natural halite (rock-salt) [27], estimating the response by fading time [28], stimating the irradiation date out of the TL GC [29], and obtaining the TL dosimetric properties of calcite obtained from nature [30], and lastly there have even been efforts to utilize machine learning methods to detect different kinds of anomalies in thermoluminescent dosimeter (TLD) glow curves [31,32,33,34,35]. Other research tried to predict the GC distortion pattern due to the variations in the TTP [36] and utilize them to determine corrected TL counts from anomalous GCs as if their shape were normal.

However, none of these methods have culminated in a comprehensive application that integrates both artificial intelligence (AI) techniques and a complementary classical deterministic algorithm to fully filter and classify glow curve anomalies and propose some means of automatic corrections when possible.

**Figure 2:**
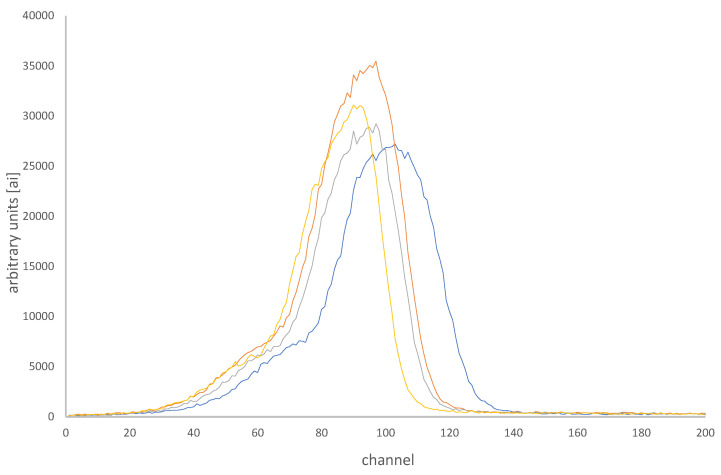
Examples of four different glow curves that are considered normal, which were filtered out by the AI filter, which uses a deep learning artificial neural network (ANN). One can see that even though the four GCs are not identical, they still have a similar shape that the ANN could capture as normal.

Good quality GCs have a unique characteristic shape (Figure 2), while anomalous or extremely low-dose GCs are mostly distinguished by their abnormal shape [37,38]. The specific shape anomalies of GCs depend on various factors such as organic contamination of the polytetrafluoroethylene (PTFE) cover on the TLD element, which may arise from dirty fingers, aging of the PTFE foils causing them to separate and to transfer heat less efficiently to the TLD element, nitrogen flow problems during hot-gas heating of the TLDs, different pre- and post-irradiation annealing procedures, the exact position of the TLD element between the PTFE foils that was set during the TLD card mounting, static electricity, electrical network interruptions, and more [24,39].

Since some hardware malfunctions of the TLD reader may result in certain GC anomalies, the detection of such anomalies may assist in identifying in advance suspicious GC shape trends that can indicate hardware problems [31,35]. The specific shape and characteristics of GCs depend on various parameters. By carefully analyzing anomalies in GCs, it is possible to detect and identify troubling trends that may signal real hardware issues. Identifying these trends in advance might prevent a decline in the EDL performance and consequently improve the accuracy of occupational dose estimation.

**Figure 3:**
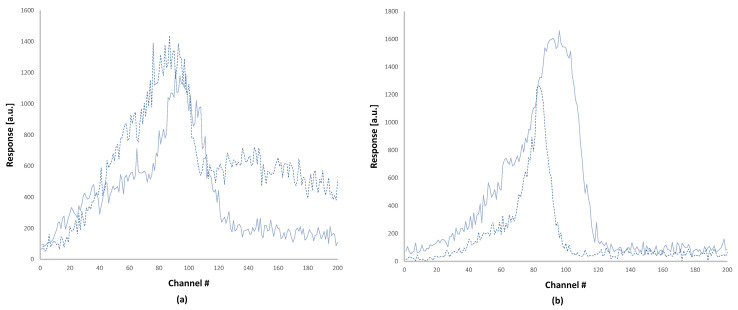

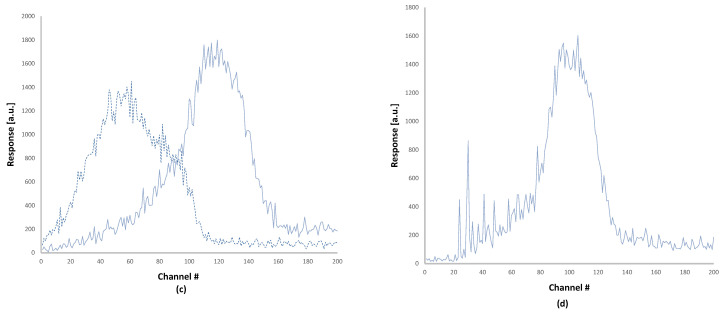
Examples for different glow curve anomaly classes: (**a**) two examples (Low and High) for class A, (**b**) two examples (Wide and Narrow) for class B, (**c**) two examples (Low and High) for class C, and (**d**) an example for class D, having spikes. The y axes of all sub-figures are in arbitrary units, and the x axes are the channel number.

Specifically, the identification of all classes but class C can give the laboratory signs regarding possible hardware problems that may arise in the near future:

− Class A Low (Figure 3a) is characterized by a high TL signal at low temperatures. Possible reasons causing this anomaly include organic (usually carbon) contamination of the Teflon cover on the TLD element from user’s dirty fingers; aging of the Teflon foils, which causes them to split from the TLD element and to transfer heat less efficiently to the TLD element; nitrogen flow problems during the heating of the TLD elements by hot-gas; and residual radiation from a prior exposure to a relatively high dose.
− Class A High (Figure 3a) is characterized by a high TL signal at high temperatures. Some possible reasons causing this anomaly include pre- and post-irradiation annealing procedures, TLD heating rate issues (GPs move to higher temperatures as the heating rate increases), and spectral response of either the photocathode or other optics such as IR filters.
− Class B Wide (Figure 3b) is characterized by wide GPs compared to normal GPs. Some possible reasons for this anomaly include TLD batch characteristics; the position of the TLD element between the Teflon foils that was set in the factory; and aging of the Teflon foils, which causes them to split from the TLD element and to transfer heat less efficiently to the TLD element.
− Class D Spikes (Figure 3d) is characterized by spikes over the GC. These spikes may be caused by static electricity (especially in TLD readers, which are located in extremely low-humidity areas); light emanates from burning particles, which arise from contamination on the TLD sample surface or on the Teflon foils from either dust or oily fingerprints; electrical network interruptions; or reader electronic interferences.

On top of automatically classifying the anomalous GCs, the TLDetect application also suggests an automatic correction in some cases. Dosimeters with GCs that cannot be corrected by TLDetect are presented to the laboratory technician for specific examination and manual correction if needed. As the categorization of GC anomalies is a subjective task (there is no single clear definition for describing these anomalies), we had to carefully define some typical anomaly classes and develop the appropriate mathematical conditions and constraints that characterize these anomalies. The classes that we defined for the possible anomalies are detailed in Table 1. Examples of all anomaly classes, which are listed in Table 1, are shown in Figure 3.

### TLDetect SW and Pipeline

The current process of examining and correcting GCs is a tedious process that consumes precious laboratory technician time each month. After the examination, the data of the GCs are entered into the Thermo © WinAlgo algorithm, which outputs the skin dose Hp(0.07) and penetrating dose Hp(10) of each dosimeter, which are the ionizing radiation dose equivalents penetrated into a depth of 0.07 mm and into a depth of 10 mm under the skin, respectively.

The newly developed TLDetect application is planned to replace the current one. TLDetect connects to the database of the Thermo © readers (TLD Harshaw 8800, Thermo Fisher Scientific Inc., Waltham, MA, USA), filters out all good quality GCs using an AI filter, and automatically classifies the GCs into the anomaly classes while also enabling an automatic correction for the class A anomaly, significantly reducing the long manual process currently carried out in the lab each month. TLDetect is also expected to improve the repeatability and accuracy of the examination of the GCs, reduce human errors, shorten the training processes in the laboratory, and save about 50 monthly work hours.

## 2. Materials and Methods

**Figure 4:**
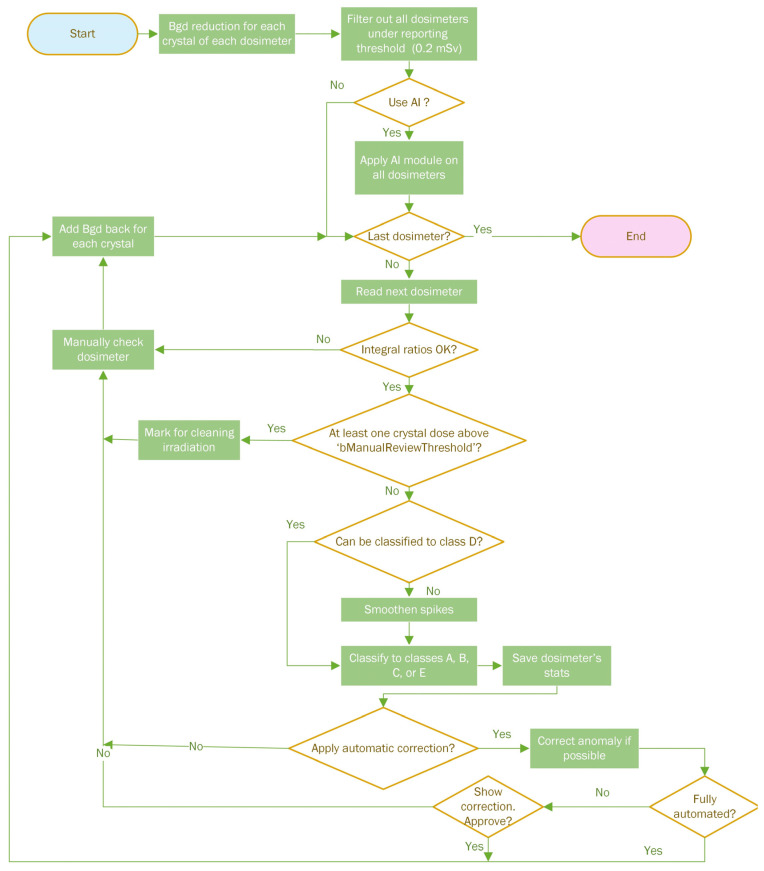
The algorithm’s high-level flow chart. The purple parts are optional stages for the user’s choice. The bold numbers at the beginning of some chart stages are references for detailed explanations of these stages in the coming section.

The algorithm’s high-level description is shown in Figure 4. In Table 2, we show a list of all algorithm parameters, their meanings, and their data types. The TLDetect application has been characterized and developed using all necessary parameters for adjusting each of the sub-algorithms that classify the GCs into each class, for defining the formulas in each, and for controlling the transition between the different stages of the algorithm.

### Detailed Algorithm Stages Review

All the referenced stages of the TLDetect algorithm block diagram, which is shown in high-level view in Figure 4, are explained in detail in the following sub-sections. Additionally, for the associated equations when denoting $Ch_{i}$, we refer to the value of the GC at channel i.

i. Background reduction

This stage is referenced in Figure 4 by the digit 1. The reduction amount is calculated by the following:

(1) $$Bgd=\frac{\mathrm{radiationPerWeek}\cdot \left[\mathrm{day}2-\mathrm{day}1\right]}{7}$$

where ‘*Bgd*’ is the calculated background radiation to be subtracted, ‘radiationPerWeek’ is a configuration file parameter that is set to 1 [mrem/week], and (day2 − day1) is the number of days that passed between the former measurement of the dosimeter and the current one. If the value turns out to be negative, we set the value to 1 mrem. This way, ‘*Bgd*’ accumulates the expected background ionizing radiation over the desired period of time, where the weekly accumulation is 1 mrem. The ‘*Bgd*’ value is saved per dosimeter in order to add it back when needed in the next filtering stage.

ii. Filtering out low-dose dosimeters

This stage is referenced in Figure 4 by the digit 2. After background reduction, TLDetect filters out all dosimeters that are under the reporting level, meaning those dosimeters whose crystals (either 3 or 4 elements) accumulated a dose of at most 16 mrems after background reduction; that is, their total dose would be less than the 20 mrems reporting level.

iii. AI Filter for normal GCs

After filtering out low-dose dosimeters, TLDetect uses a pre-trained deep learning ANN in order to filter out all dosimeters that have a regular shape. All the remaining dosimeters, which are suspected as anomalous GCs, are directed to the next filters. Examples of regular GCs that were filtered by the TLDetect algorithm are shown in Figure 2.

The ANN was trained using a total of 1608 different GCs; 144 of them were anomalous GCs, and the rest were different examples of normal GCs. The network itself contains 3 inner layers of depths 5, 15, and 5 nodes each. The input layer contains 202 nodes: one node for each of the 200 channels and 2 more inputs—one for the kurtosis and the second for the skewness of the GCs—both of which are good features that contributed to the ability of the algorithm to classify anomalous GCs as they represent measures of the distribution shape. The output of the ANN is a single number encompassing the probability of the GC being a normal one. The more the probability is closer to 1, the more the GC’s shape is similar to that of a normal GC. We used 60% of the labeled GCs for the training process, 20% for the validation, and the last 20% for testing. It is interesting to note that we chose a low decision threshold for normal GC classification at a value of 0.91. We chose this number since we preferred that the filter would give us false negative classifications (the algorithm classifies normal GCs as anomalous ones) overthe possibility of the filter to give us false positive classifications (the algorithm classifies anomalous GCs as normal ones).

iv. Integral ratios filter

This stage is referenced in Figure 4 by the digit 3. Using two conditions, it checks that all 3 or 4 crystal doses lie inside element ratios that reflect possible radiation fields [5], and only in this case, TLDetect continues to the next stages of the classification. The first condition is applied using Equation (2). If it is true, then algorithm passes to the next stage; if it is false, then the second condition is checked using Equation (3), where each crystal triplet or quadrat should match at least one line in Table 3. If the second condition is met, then the algorithm passes to the next stage; if it is false, then TLDetect classifies this dosimeter for manual inspection.

(2) $$\mathrm{Max}\left(L1,L2,L3,L4\right)-\mathrm{Min}\left(L1,L2,L3,L4\right)/\mathrm{Max}\left(L1,L2,L3,L4\right)<\mathrm{Crystals}\_\mathrm{Quotient}$$

(3) 3 or 4 ratios of crystal doses match a row in table 3 upto ±ratio_table_threshold

where both Crystals_Quotient and ratio_table_threshold are parameters defined inside the configuration file.

v. Class D classification smoothening

This stage is referenced in Figure 4 by the digit 4. TLDetect first tries to classify the GCs into class D, which is the spikes class (Figure 3d).

If the GC is classified to class D, then the algorithm will not try to classify it to another class and will continue to the next GC. On the other hand, if the algorithm cannot classify the GC to class D, it will smoothen it before continuing to the next filters. Experience shows that spikes mostly have either one-channel or two-channels width. In order to check for spikes, the algorithm checks Equation (4) with its three conditions—the first condition must be satisfied and either of the second or third one—for all channels i from 1 to 197:

(4) Chi+1>Chi·100+SpikesNeighDiff100And Chi+1>Chi+2·100+SpikesNeighDiff100Or Chi+2>Chi+3·100+SpikesNeighDiff100

where ${\mathrm{Ch}}_{i}$ is the value of GC at channel i and ‘SpikesNeighDiff’ is a configuration file parameter. If TLDetect finds more than ‘NSpikes’ spikes satisfying Equation (4) conditions, then the GC is classified to class D. Otherwise, it will move on to the next stage of the algorithm for smoothening.

vi. Smoothening

This stage is referenced in Figure 4 by the digit 5. If TLDetect was not able to classify the GC to class D, TLDetect will first smoothen it before trying to classify it to all other classes. The difference between a GC that was classified to class D and one which was smoothened for spikes is that the former one has at least ‘Nspikes’ (defined in the configuration file, as shown in Table 2) spikes, while the latter one will have less than ‘Nspikes’ spikes, allowing it to be smoothened and then to be more easily handled by other classifiers. After smoothening, the GC moves on to the next algorithm stage of trying to classify it to either the A, B, C, or E class.

vii. Classify GC to either Class A, B, C or E

This stage is referenced in Figure 4 by the digit 6. It actually contains the classification process to three different classes or to the last-resort anomaly class E if the GC could not be classified to either of classes A, B, or C. All four classes A, B, C, and E are exclusive, which means that a GC can only be classified to one of them at a time. On the other hand, a GC can be simultaneously classified to both class A Low and class A High.

a. Class A classification

To classify a GC to class A (Figure 3a), the algorithm first validates that the maximal GC value at ${\mathrm{Ch}}_{max}$ lies inside the channel interval 95 ± MaxAllowedShift, where ‘MaxAllowedShift’ is a configuration file parameter defined in Table 2. Afterwards, if both conditions of inequality in Equation (5) are satisfied, then the GC is classified into class A, sub-class High.

(5) AvgChBgdHTTLChLow… ChBgdHTTLChHigh<0.01·MaxBgdHeight·Max And  AvgChBgdHTTLChLow… ChBgdHTTLChHigh>0.01·MinBgdHeight·Max

(6) AvgChBgdLTTLChLow… ChBgdLTTLChHigh<0.01·MaxBgdHeight·Max And  AvgChBgdLTTLChLow… ChBgdLTTLChHigh>0.01·MinBgdHeight·Max

If, on the other hand, both conditions of inequality of Equation (6) are satisfied, then the GC is classified into class A, sub-class Low. It is easy to see that by definition a GC can be simultaneously classified to both sub-classes of class A. In any case, either satisfying Equation (5) or satisfying Equation (6), TLDetect moves on to the next dosimeter while marking the current dosimeter as a class A dosimeter. If neither Equation (5) nor Equation (6) is satisfied, then the algorithm continues with the dosimeter to class C classification.

b. Class C classification

To classify a GC to class C (Figure 3c), the algorithm finds the maximal GC value at channel ${\mathrm{Ch}}_{max}$. If Equation (7) is satisfied, then the GC is classified to class C, sub-class High. If, on the other hand, Equation (8) is satisfied, then the GC is classified into class C, sub-class Low.

(7) $${\mathrm{Ch}}_{max}>95+\mathrm{MaxAllowedShift}$$

(8) $${\mathrm{Ch}}_{max}<95-\mathrm{MaxAllowedShift}$$

In any case, either satisfying Equation (7) or satisfying Equation (8), TLDetect moves on to the next dosimeter while marking the current dosimeter as a class C dosimeter. If neither Equation (7) nor Equation (8) is satisfied, then the algorithm continues with the dosimeter to class B classification.

c. Class B classification

To classify a GC to class B (Figure 3b), the algorithm first validates that the maximal GC value at ${\mathrm{Ch}}_{max}$ lies inside the channel interval 95 ± MaxAllowedShift, where ‘MaxAllowedShift’ is a configuration file parameter defined in Table 2. Afterwards, if the inequality in Equation (9) is satisfied, then the dosimeter is classified into class B, sub-class WIDE, and TLDetect moves on to the next dosimeter. In Equation (9), Avg stands for the average value, and Max is the maximal value of the GC.

(9) $$Avg\left({\mathrm{Ch}}_{WideLowCh}\ldots {\;\mathrm{Ch}}_{WideHighCh}\right)>0.01\cdot \mathrm{WideAvgVal}\cdot \mathrm{Max}$$

If, on the other hand, both conditions of inequality in Equation (10) are satisfied, then the dosimeter is classified into class B, sub-class NARROW, and TLDetect moves on to the next dosimeter.

(10) AvgCh1… ChMax− half_width_num_ch<0.01·NarrowAvgVal·Max And AvgChMax+ half_width_num_ch… Ch200<0.01·NarrowAvgVal·Max

If the GC was classified into neither of the class B sub-classes, then the algorithm continues with the dosimeter to class E classification.

d. Class E classification

To classify a GC to class E, the only conditions that need to be fulfilled are conditions ii, filtering out low-dose dosimeters, and iii, integral ratio filters, along with unsuccessful classification to A, B, C, and D classes. This means that once a GC was diagnosed as an anomalous one, but could not be classified to any of these four classes, it will be classified to the default E anomalous class.

viii. Anomaly correction

This stage is referenced in Figure 4 by the digit 7. The anomaly correction is currently possible only for GCs that were classified to class A, sub-classes Low or High. This anomaly class is easy and straightforward to correct by reducing the background dose at either the low or the high temperature channel. TLDetect will offer the technician its automatic suggestion for correcting the GC. If the configuration file parameter ‘bFullAutomated’ equals true, then TLDetect will automatically correct the GC without the need for technician approval; otherwise, TLDetect will suggest a correction that will need to be approved by the technician before taking place.

## 3. Results and Discussion

### 3.1. Business Intelligence Tool

As a complimentary tool for the TLDetect application, we also developed a business intelligence (BI) tool based on QlikView© SW [40]. This tool allows the TLDetect user, i.e., the EDL technicians, to run complex database queries over a broad range of parameters and to present their results in a clear manner.

TLDetect online saves a comprehensive database of all processed dosimeters, including their complete statistics, which includes minimal, maximal, and median values of their GCs, their element correction coefficients (ECC), their responses, the anomaly classes they were classified to, all the filters and thresholds they passed/failed, time stamps, and more.

### 3.2. Results

**Figure 5:**
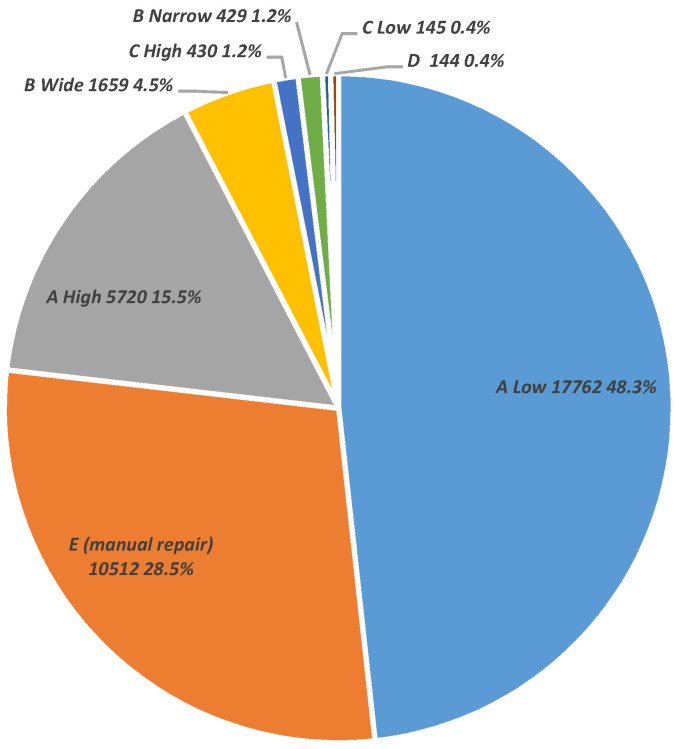
A distribution example produced using the Qlikview BI helping tool. Out of the 97,512 TLDs that were analyzed, 58,550 were under the 16 mrem threshold. From the remaining 38,962 TLDs, the AI filter found 7696 good TLDs, and the rest of them—31,266 dosimeters—were classified into the shown anomaly classes. Some of the TLDs can be simultaneously classified to both A Low and A High classes.

Some interesting results of the TLDetect application are shown in Figure 5. We ran TLDetect over a set of 97,512 different GCs that were measured at our lab from 2020 to 2023 and analyzed its filters and classification results. As expected, since most of the GCs belong to the EDL’s customers, which are rarely exposed to high doses of ionizing radiation, 58,500 dosimeters were filtered out as not crossing the Israeli reporting ionizing radiation threshold, which takes the value of 0.2 mSv. Out of the remaining dosimeters whose results surpassed the reporting threshold, the AI module filtered 19.8% of them out, so an amount of 31,266 dosimeters continued on to the next filters.

The complementary BI tool we developed will supply detailed information that will be kept for years ahead and will enable the EDL to easily analyze dosimetric information and trends in time. These trends might imply hardware issues in the TLD reader or the dosimeter itself and assist in predicting problems that might occur in TLD readers, such as electronic noise, which may cause GC spikes, or aging of the Teflon cover on the TLD element, which may cause a high signal in the low-temperature channels (class A LOW).

## Figures and Tables

**Table 1:** The different anomaly classes of the glow curves.

Anomaly Class	Sub Class	Definition
Class A	Low	High background TL signal at low temperatures
	High	High background TL signal at high temperatures
Class B	Wide	Invalid GC width—too wide a GC
	Narrow	Invalid GC width—too narrow a GC
Class C	Low	GC shifted towards low temperatures
	High	GC shifted towards high temperatures
Class D	-	Too many spikes

**Table 2:** All algorithm parameters inside the configuration file.

Parameter Name	Class/Module	Description	Units
ManualReviewThreshold	General	Above this value, a dosimeter will be manually checked	mrem
bFullAutomated		Correct automatically without approval or not	bool
Threshold1		Threshold under which 1st crystal is filtered out	mrem
Threshold2		Threshold under which 2nd crystal is filtered out	
Threshold3		Threshold under which 3rd crystal is filtered out	
ThresholdNeut		Threshold under which 4th crystal (thermal neutrons crystal) is filtered out	
ThresholdRing		Threshold under which ring crystal is not checked	
RadiationPerWeek		Weekly background	
Crystals_Quotient		Ratio filter factor #1	0–1
ratio_table_threshold		Ratio filter factor #2	0–1
MDBPath		File path for winrems SQL data	string
bUseAI	Machine Learning	Use AI filter	bool
training_model_file		ANN Model file name	-
ai_probability_threshold		Above this threshold, GC is classified as normal	-
MaxBgdHeight	Class A	Bgd max height relative to GC max height	%
MinBgdHeight		Bgd min height relative to GC max height	
BgdHTTLChLow		Minimal channel of high temperature	-
BgdHTTLChHigh		Maximal channel of high temperature	-
BgdLTTLChLow		Minimal channel of low temperature	-
BgdLTTLChHigh		Maximal channel of low temperature	-
LowCutCh		Max channel index for A_LTTL cut	-
HighCutCh		Min channel index for A_HTTL cut	-
TLDWideLowCh	Class B	Minimal channel for width measure	-
TLDWideHighCh		Maximal channel for width measure	-
WideAvgVal		Average height between minimal and maximal channels relative to max GC height	%
half_width_num_ch		Half-width of narrow GC in numberof channels unit	-
NarrowAvgVal		GC height relative to max height outside the narrow channels	%
MaxAllowedShift		Max allowed shift from channel 95	-
NSpikes	Class D	Number of spikes found in GC	-
SpikeNeighDiff		Percent difference between two neighbor channels	%

**Table 3:** Integral ratio check table. Each beam name is an abbreviation for a different kind of radiation field. The different beam abbreviation stands for different kinds of energy beams or radioisotopes.

L4/L3	L3/L1	L3/L2	L1/L4	E (keV)	Beam
0.8	7.80	2.10	0.15	20	NS 25
0.92	3.19	1.47	0.34	24	NS30
0.95	1.50	1.15	0.70	33	NS40
0.96	1.04	1.03	1.00	48	NS60
0.97	0.94	0.99	1.09	65	NS80
0.97	0.93	1.00	1.11	83	NS100
0.98	0.96	1.00	1.06	100	NS120
0.96	0.96	1.01	1.09	118	NS150
0.98	0.94	1.01	1.09	164	NS200
0.97	0.93	1.03	1.10	208	NS250
0.97	0.89	0.97	1.16	250	NS300
1.02	0.97	1.03	1.01	118	H150
0.79	8.88	2.90	0.14	20	M30
0.93	1.96	1.35	0.55	35	M60
0.96	1.14	1.08	0.92	53	M100
0.98	0.97	1.01	1.05	73	M150
0.96	1.41	1.15	0.74	38	S60
1.00	1.00	1.00	1.00	662	Cs137
0.00	6200	6200	0.05		Tl204
0.30	4.98	67.5	0.68		Sr90/Y90
0.23	6.44	24.2	0.69		DU

## Data Availability

Data available on request due to restrictions of customers health privacy.

## References

[1] Shubayr N., Alashban Y., Almalki M., Aldawood S., Aldosari A. (2021). Occupational radiation exposure among diagnostic radiology workers in the Saudi ministry of health hospitals and medical centers: A five-year national retrospective study. J. King Saud Univ. Sci..

[2] Zhang G., Shen J., Bao P., Yao Z., Yuan Y., Fang S. (2020). Assessment of occupational exposure of radiation workers at a tertiary hospital in Anhui province, China, during 2013–18. Radiat. Prot. Dosim..

[3] Baudin C., Vacquier B., Thin G., Chenene L., Guersen J., Partarrieu I., Louet M., Ducou Le Pointe H., Mora S., Verdun-Esquer C. (2023). Occupational exposure to ionizing radiation in medical staff: Trends during the 2009–2019 period in a multicentric study. Eur. Radiol..

[4] Wilson-Stewart K.S., Fontanarosa D., Malacova E., Trapp J.V. (2023). A comparison of patient dose and occupational eye dose to the operator and nursing staff during transcatheter cardiac and endovascular procedures. Sci. Rep..

[5] Richardson D.B., Leuraud K., Laurier D., Gillies M., Haylock R., Kelly-Reif K., Bertke S., Daniels R.D., Thierry-Chef I., Moissonnier M. (2023). Cancer mortality after low dose exposure to ionising radiation in workers in France, the United Kingdom, and the United States (INWORKS): Cohort study. BMJ.

[6] Omer H., Salah H., Tamam N., Mahgoub O., Sulieman A., Ahmed R., Abuzaid M., Saad I.E., Almogren K.S., Bradley D.A. (2023). Assessment of occupational exposure from PET and PET/CT scanning in Saudi Arabia. Radiat. Phys. Chem..

[7] Boice J.D., Cohen S.S., Mumma M.T., Howard S.C., Yoder R.C., Dauer L.T. (2022). Mortality among medical radiation workers in the United States, 1965–2016. Int. J. Radiat. Biol..

[8] Cha E.S., Zablotska L.B., Bang Y.J., Lee W.J. (2020). Occupational radiation exposure and morbidity of circulatory disease among diagnostic medical radiation workers in South Korea. Occup. Environ. Med..

[9] Alkhorayef M., Mayhoub F.H., Salah H., Sulieman A., Al-Mohammed H.I., Almuwannis M., Kappas C., Bradley D.A. (2020). Assessment of occupational exposure and radiation risks in nuclear medicine departments. Radiat. Phys. Chem..

[10] Zhou J., Li W., Deng J., Li K., Jin J., Zhang H. (2023). Trend and distribution analysis of occupational radiation exposure among medical practices in Chongqing, China (2008–2020). Radiat. Prot. Dosim..

[11] Garcia-Sayan E., Jain R., Wessly P., Mackensen G.B., Johnson B., Quader N. (2024). Radiation Exposure to the Interventional Echocardiographers and Sonographers: A Call to Action. J. Am. Soc. Echocardiogr..

[12] Azizova T.V., Bannikova M.V., Briks K.V., Grigoryeva E.S., Hamada N. (2023). Incidence risks for subtypes of heart diseases in a Russian cohort of Mayak Production Association nuclear workers. Radiat. Environ. Biophys..

[13] Ikezawa K., Hayashi S., Takenaka M., Yakushijin T., Nagaike K., Takada R., Yamai T., Matsumoto K., Yamamoto M., Omoto S. (2023). Occupational radiation exposure to the lens of the eyes and its protection during endoscopic retrograde cholangiopancreatography. Sci. Rep..

[14] Al-Haj A.N., Lagarde C.S. (2004). Glow curve evaluation in routine personal dosimetry. Health Phys..

[15] Horowitz Y.S., Moscovitch M. (2013). Highlights and pitfalls of 20 years of application of computerised glow curve analysis to thermoluminescence research and dosimetry. Radiat. Protect. Dosim..

[16] Horowitz Y.S., Yossian D. (1995). Computerized glow curve deconvolution applied to the analysis of the kinetics of peak 5 in LiF: Mg,Ti (TLD-100). J. Phys. Appl. Phys..

[17] Horowitz Y.S., Yossian D. (1995). Computerised glow curve deconvolution: Application to thermoluminescence dosimetry. Radiat. Protect. Dosim..

[18] Karmakar M., Bhattacharyya S., Sarkar A., Mazumdar P.S., Singh S.D. (2017). Analysis of thermoluminescence glow curves using derivatives of different orders. Radiat. Protect. Dosim..

[19] Sadek A.M., Farag M.A., El-Hafez A.I.A., Kitis G. (2024). TL-SDA: A designed toolkit for the deconvolution analysis of thermoluminescence glow curves. Appl. Radiat. Isot..

[20] Peng J., Kitis G., Sadek A.M., Karsu Asal E.C., Li Z. (2021). Thermoluminescence glow-curve deconvolution using analytical expressions: A unified presentation. Appl. Radiat. Isot..

[21] Stadtmann H., Wilding G. (2017). Glow curve deconvolution for the routine readout of LiF: Mg,Ti. Radiat. Meas..

[22] Osorio P.V., Stadtmann H., Lankmayr E. (2001). A new algorithm for identifying abnormal glow curves in thermoluminescence personal dosimetry. Radiat. Protect. Dosim..

[23] Osorio P.V., Stadtmann H., Lankmayr E. (2002). An example of abnormal glow curves identification in personnel thermoluminescent dosimetry. J. Biochem. Biophys. Methods.

[24] Pradhan S.M., Sneha C., Adtani M.M. (2011). A method of identification of abnormal glow curves in individual monitoring using CaSO4:Dy teflon TLD and hot gas reader. Radiat. Prot. Dosim..

[25] Moscovitch M., Rotunda J.E., Tawil R.A., Rathbone B.A. (1995). A TLD dose algorithm using artificial neural networks. Radioact. Radiochem..

[26] Mentzel F., Derugin E., Jansen H., Kröninger K., Nackenhorst O., Walbersloh J., Weingarten J. (2021). No more glowing in the dark: How deep learning improves exposure date estimation in thermoluminescence dosimetry. J. Radiol. Prot..

[27] Toktamis D., Er M.B., Isik E. (2022). Classification of thermoluminescence features of the natural halite with machine learning. Radiat. Eff. Defects Solids.

[28] Kröninger K., Mentzel F., Theinert R., Walbersloh J. (2019). A machine learning approach to glow curve analysis. Radiat. Meas..

[29] Mentzel F., Kröninger K., Röhrig L., Speicher L., Steil M.L., Theinert R., Walbersloh J. (2020). Extending information relevant for personal dose monitoring obtained from glow curves of thermoluminescence dosimeters using artificial neural networks. Radiat. Meas..

[30] Isik E. (2022). Thermoluminescence characteristics of calcite with a Gaussian process regression model of machine learning. Luminescence.

[31] Pathan M.S., Pradhan S.M., Selvam T.P., Sapra B.K. (2023). A machine learning approach for correcting glow curve anomalies in CaSO_4_: Dy-based TLD dosimeters used in personnel monitoring. J. Radiol. Prot..

[32] Pathan M.S., Pradhan S.M., Palani Selvam T. (2020). Machine learning algorithms for identification of abnormal glow curves and associated abnormality in CaSO4: Dy-based personnel monitoring dosimeters. Radiat. Prot. Dosim..

[33] Amit G., Datz H. (2018). Improvement of Dose Estimation Process Using Artificial Neural Networks. Radiat. Prot. Dosim..

[34] Amit G., Datz H. (2018). Automatic detection of anomalous thermoluminescent dosimeter glow curves using machine learning. Radiat. Meas..

[35] Amit G., Datz H. (2019). Computerized Categorization of TLD Glow Curve Anomalies Using Multi-Class Classification Support Vector Machines. Radiat. Meas..

[36] Pathan M.S., Pradhan S.M., Datta D., Selvam T. (2019). P Study of effect of consecutive heating on thermoluminescence glow curves of multi-element TL dosemeter in hot gas-based reader system. Radiat. Prot. Dosim..

[37] Sadek A.M., Abdou N.Y., Alazab H. (2022). A Uncertainty of LiF thermoluminescence at low dose levels: Experimental results. Appl. Radiat. Isot..

[38] Sadek A.M. (2020). Uncertainty of thermoluminescence at low dose levels: A Monte-Carlo simulation study. Radiat. Protect. Dosim..

[39] Horowitz Y.S., Oster L., Datz H. (2007). The thermoluminescence dose–response and other characteristics of the high-temperature TL in LiF: Mg,Ti (TLD-100). Radiat. Protect. Dosim..

[40] QlikTech International AB (2011). Business Discovery: Powerful, User-Driven BI: A QlikView White Paper. https://www.retailitinsights.com/doc/business-discovery-powerful-user-driven-bi-0002.

